# Piperazinediium tetra­chloridocadmate monohydrate

**DOI:** 10.1107/S1600536811005095

**Published:** 2011-02-16

**Authors:** Meher El Glaoui, Imen Ben Gharbia, Valeria Ferretti, Cherif Ben Nasr

**Affiliations:** a Laboratoire de Chimie des Matériaux, Faculté des Sciences de Bizerte, 7021 Zarzouna, Tunisia; b Chemistry Department and Centro di Strutturistica Diffrattometrica, University of Ferrara, Via L. Borsari 46, I-44121 FerrarA, Italy

## Abstract

In the title compound, (C_4_H_12_N_2_)[CdCl_4_]·H_2_O, the [CdCl_4_]^2−^ anions adopt a slightly distorted tetra­hedral configuration. In the crystal, O—H⋯Cl hydrogen bonds link the anions and water mol­ecules into corrugated inorganic chains along the *b* axis which are inter­connected *via* piperazinediiumN—H⋯O and N—H⋯Cl inter­actions into a three-dimensional framework structure.

## Related literature

For common applications of organic–inorganic hybrid mat­erials, see: Kobel & Hanack (1986 ▶); Pierpont & Jung (1994 ▶). For a related structure and discussion of geometrical features, see: Sutherland & Harrison (2009 ▶). For the coordination around the Cd^II^ cation, see: El Glaoui *et al.* (2009 ▶).
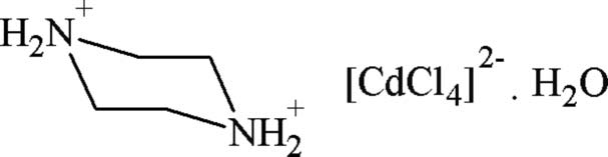


## Experimental

### 

#### Crystal data

(C_4_H_12_N_2_)[CdCl_4_]·H_2_O
*M* *_r_* = 360.38
Monoclinic, 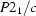

*a* = 6.6204 (2) Å
*b* = 12.8772 (3) Å
*c* = 14.0961 (4) Å
β = 92.1710 (12)°
*V* = 1200.86 (6) Å^3^
*Z* = 4
Mo *K*α radiation
μ = 2.67 mm^−1^
*T* = 295 K
0.52 × 0.48 × 0.30 mm

#### Data collection

Nonius KappaCCD diffractometer
Absorption correction: multi-scan (*SORTAV*; Blessing, 1995 ▶) *T*_min_ = 0.374, *T*_max_ = 0.444
8531 measured reflections
3461 independent reflections
2903 reflections with *I* > 2σ(*I*)
*R*_int_ = 0.037

#### Refinement

*R*[*F* ^2^ > 2σ(*F* ^2^)] = 0.033
*wR*(*F* ^2^) = 0.081
*S* = 1.09
3461 reflections
126 parameters
H atoms treated by a mixture of independent and constrained refinement
Δρ_max_ = 0.78 e Å^−3^
Δρ_min_ = −1.75 e Å^−3^

### 

Data collection: *Kappa-CCD Server Software* (Nonius, 1997 ▶); cell refinement: *DENZO-SMN* (Otwinowski & Minor, 1997 ▶); data reduction: *DENZO-SMN*; program(s) used to solve structure: *SIR97* (Altomare *et al.*, 1999 ▶); program(s) used to refine structure: *SHELXL97* (Sheldrick, 2008 ▶); molecular graphics: *ORTEPIII* (Burnett & Johnson, 1996 ▶); software used to prepare material for publication: *SHELXL97* and *WinGX* (Farrugia, 1999 ▶).

## Supplementary Material

Crystal structure: contains datablocks global, I. DOI: 10.1107/S1600536811005095/zs2095sup1.cif

Structure factors: contains datablocks I. DOI: 10.1107/S1600536811005095/zs2095Isup2.hkl

Additional supplementary materials:  crystallographic information; 3D view; checkCIF report

## Figures and Tables

**Table 1:** Hydrogen-bond geometry (Å, °)

*D*—H⋯*A*	*D*—H	H⋯*A*	*D*⋯*A*	*D*—H⋯*A*
N1—H1⋯Cl1^i^	0.93 (3)	2.35 (3)	3.254 (2)	164 (3)
N1—H2⋯Cl3	0.89 (3)	2.41 (4)	3.155 (2)	141 (3)
N2—H3⋯O1*W*	0.89 (3)	1.93 (3)	2.808 (3)	167 (3)
N2—H4⋯Cl4^ii^	0.81 (3)	2.46 (3)	3.190 (2)	151 (3)
O1*W*—H1*W*⋯Cl2^iii^	0.84	2.44	3.267 (3)	168
O1*W*—H2*W*⋯Cl4^iv^	0.85	2.54	3.304 (2)	150

Symmetry codes: (i) 
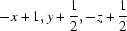
; (ii) 
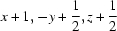
; (iii) 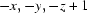; (iv) 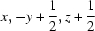.

**Fig. 1.**
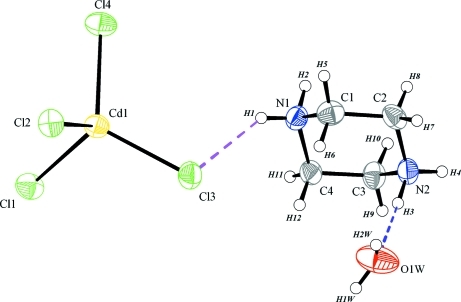
A view of the title compound, showing 50% probability displacement ellipsoids and the atom numbering scheme. Dashed lines indicate N—H···O and N—H···Cl hydrogen bonds.

**Fig. 2.**
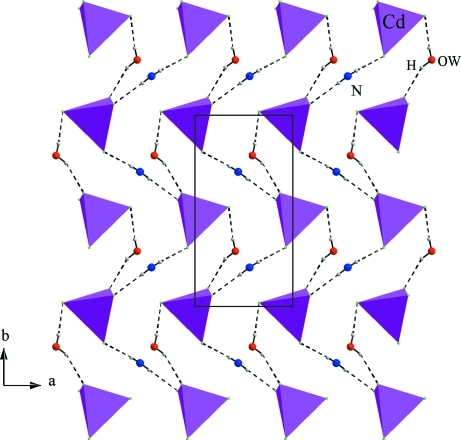
A projection along the c axis of the inorganic layer structure at z = 1/4.

**Fig. 3.**
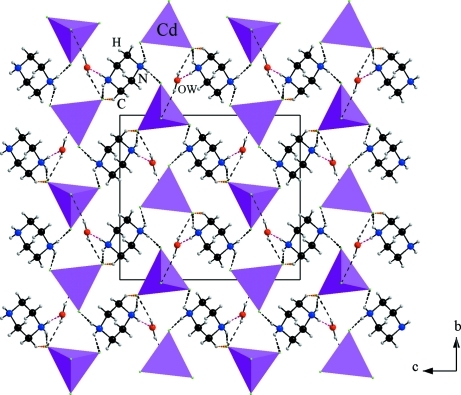
The packing of the title compound viewed down the a axis. Hydrogen bonds are shown as dotted lines.

## supplementary crystallographic information

### Comment

Organic-inorganic hybrid materials continue to attract much attention due to their potential applications in various field (Kobel & Hanack, 1986; Pierpont & Jung, 1994). In this work, we report the crystal structure of one such compound, C_4_H_12_N_2_ [CdCl_4_] . H_2_O (I), formed from the reaction of piperazine with cadmium chloride. In (I) the asymmetric unit comprises a piperazine-1,4-diium dication, a [CdCl_4_]^2-^ anion and a water molecule of solvation (Fig. 1). The atomic arrangement of (I) can be described as built up of corrugated inorganic chains of [CdCl_4_]^2-^ tetrahedra and water molecules held together by O—H···Cl hydrogen bonds and extending along the *b* direction of the unit cell. These chains are interconnected by a set of piperazinium N—H···Cl hydrogen bonds to form layers extending along the (1 1 O) planes (Fig. 2, Table 1). Fig 3 shows that two such layers cross the unit cell at *z* = 1/4 and *z* = 3/4 and the bodies of the organic groups are located between these layers and connect them by weak C—H···Cl hydrogen bonds [C···Cl, 3.535 (3) Å], giving a three-dimensional framework structure. In the organic entity, the piperazium ring adopts a typical chair conformation and all the geometrical features agree with those found in piperazindiium tetrachlorozincate(II) (Sutherland & Harrison, 2009). It is worth noting that in the anion of (I), the Cd—Cl bond lengths and Cl—Cd—Cl bond angles are not equal, but vary with the environment around the Cl atom. The Cd—Cl bond lengths vary between 2.4418 (6) and 2.4892 (7) Å and the Cl—Cd—Cl angles range from 103.07 (2) to 115.19 (2) °. These values are in good agreement with those reported previously, clearly indicating that the [CdCl_4_]^2-^ anion has a slightly distorted tetrahedral stereochemistry (El Glaoui *et al.* (2009).

### Experimental

An aqueous solution of piperazine (4 mmol, 0.344 g), cadmium chloride (4 mmol, 0.732 g) and HCl (10 ml, 0.8 *M*) in a Petri dish was slowly evaporated at room temperature. Single crystals of the title compound, suitable for X-ray diffraction analysis, were obtained after several days (yield 68%).

### Refinement

All N—H hydrogen atoms were found in the difference Fourier map and refined isotropically. The water hydrogen atoms were also found in the difference Fourier but their positions were kept fixed during the refinement and their *U*_iso_ values were given a value equal to 1.2 times *U*_eq_ of the parent oxygen. All C—H atoms were allowed to ride with C—H = 0.97 Å and *U*_iso_(H) = 1.2*U*_eq_(C).

### Crystal data

**Table:**

(C_4_H_12_N_2_)[CdCl_4_]·H_2_O	*F*(000) = 704
*M**_r_* = 360.38	*D*_x_ = 1.993 Mg m^−^^3^
Monoclinic, *P*2_1_/*c*	Mo *K*α radiation, λ = 0.71073 Å
Hall symbol: -P 2ybc	Cell parameters from 8531 reflections
*a* = 6.6204 (2) Å	θ = 2.0–30.0°
*b* = 12.8772 (3) Å	µ = 2.67 mm^−^^1^
*c* = 14.0961 (4) Å	*T* = 295 K
β = 92.1710 (12)°	Prismatic, colourless
*V* = 1200.86 (6) Å^3^	0.52 × 0.48 × 0.30 mm
*Z* = 4	

### Data collection

**Table:**

Nonius KappaCCD diffractometer	3461 independent reflections
Radiation source: fine-focus sealed tube	2903 reflections with *I* > 2σ(*I*)
graphite	*R*_int_ = 0.037
φ scans and ω scans	θ_max_ = 30.0°, θ_min_ = 3.1°
Absorption correction: multi-scan (*SORTAV*; Blessing, 1995)	*h* = −9→9
*T*_min_ = 0.374, *T*_max_ = 0.444	*k* = −17→17
8531 measured reflections	*l* = −19→19

### Refinement

**Table:**

Refinement on *F*^2^	Secondary atom site location: difference Fourier map
Least-squares matrix: full	Hydrogen site location: difference Fourier map
*R*[*F*^2^ > 2σ(*F*^2^)] = 0.033	H atoms treated by a mixture of independent and constrained refinement
*wR*(*F*^2^) = 0.081	*w* = 1/[σ^2^(*F*_o_^2^) + (0.0389*P*)^2^ + 0.4362*P*] where *P* = (*F*_o_^2^ + 2*F*_c_^2^)/3
*S* = 1.09	(Δ/σ)_max_ < 0.001
3461 reflections	Δρ_max_ = 0.78 e Å^−^^3^
126 parameters	Δρ_min_ = −1.75 e Å^−^^3^
0 restraints	Extinction correction: *SHELXL97* (Sheldrick, 2008), Fc^*^=kFc[1+0.001xFc^2^λ^3^/sin(2θ)]^-1/4^
Primary atom site location: structure-invariant direct methods	Extinction coefficient: 0.0778 (19)

### Special details

**Table:**

Geometry. All esds (except the esd in the dihedral angle between two l.s. planes) are estimated using the full covariance matrix. The cell esds are taken into account individually in the estimation of esds in distances, angles and torsion angles; correlations between esds in cell parameters are only used when they are defined by crystal symmetry. An approximate (isotropic) treatment of cell esds is used for estimating esds involving l.s. planes.
Refinement. Refinement of *F*^2^ against ALL reflections. The weighted *R*-factor *wR* and goodness of fit *S* are based on *F*^2^, conventional *R*-factors *R* are based on *F*, with *F* set to zero for negative *F*^2^. The threshold expression of *F*^2^ > σ(*F*^2^) is used only for calculating *R*-factors(gt) *etc*. and is not relevant to the choice of reflections for refinement. *R*-factors based on *F*^2^ are statistically about twice as large as those based on *F*, and *R*- factors based on ALL data will be even larger.

### Fractional atomic coordinates and isotropic or equivalent isotropic displacement parameters (Å^2^)

**Table:**

	*x*	*y*	*z*	*U*_iso_*/*U*_eq_	
Cd1	0.02921 (3)	−0.001890 (11)	0.235463 (13)	0.03178 (9)	
Cl1	0.07520 (10)	−0.19208 (5)	0.22725 (4)	0.03972 (16)	
Cl2	−0.34472 (9)	0.01950 (5)	0.22883 (5)	0.03628 (15)	
Cl3	0.17473 (9)	0.05843 (5)	0.38740 (5)	0.03822 (15)	
Cl4	0.13421 (9)	0.09489 (5)	0.09660 (5)	0.03906 (16)	
N1	0.5582 (3)	0.20385 (16)	0.38723 (15)	0.0312 (4)	
N2	0.7142 (3)	0.28658 (16)	0.56419 (15)	0.0321 (4)	
C1	0.4546 (4)	0.29108 (19)	0.43473 (18)	0.0358 (5)	
H5	0.3509	0.2636	0.4746	0.043*	
H6	0.3897	0.3355	0.3871	0.043*	
C2	0.6036 (4)	0.35402 (17)	0.49466 (17)	0.0343 (5)	
H7	0.6991	0.3875	0.4540	0.041*	
H8	0.5322	0.4077	0.5281	0.041*	
C3	0.8195 (4)	0.20021 (19)	0.51588 (18)	0.0345 (5)	
H9	0.8879	0.1564	0.5629	0.041*	
H10	0.9203	0.2285	0.4749	0.041*	
C4	0.6703 (4)	0.13687 (17)	0.45821 (17)	0.0318 (5)	
H11	0.5752	0.1046	0.4998	0.038*	
H12	0.7406	0.0822	0.4255	0.038*	
O1W	0.4133 (3)	0.2110 (2)	0.68116 (18)	0.0644 (7)	
H1	0.646 (5)	0.232 (2)	0.344 (2)	0.038 (8)*	
H2	0.461 (5)	0.168 (3)	0.357 (2)	0.053 (9)*	
H3	0.632 (5)	0.255 (2)	0.604 (2)	0.040 (8)*	
H4	0.803 (5)	0.319 (2)	0.592 (2)	0.041 (8)*	
H1W	0.3888	0.1492	0.6962	0.080*	
H2W	0.3114	0.2506	0.6752	0.080*	

### Atomic displacement parameters (Å^2^)

**Table:**

	*U*^11^	*U*^22^	*U*^33^	*U*^12^	*U*^13^	*U*^23^
Cd1	0.03183 (12)	0.03025 (13)	0.03330 (13)	0.00147 (6)	0.00181 (8)	0.00206 (6)
Cl1	0.0483 (4)	0.0303 (3)	0.0411 (3)	0.0064 (2)	0.0091 (3)	−0.0011 (2)
Cl2	0.0298 (3)	0.0379 (3)	0.0412 (3)	0.0020 (2)	0.0026 (2)	−0.0031 (2)
Cl3	0.0358 (3)	0.0386 (3)	0.0400 (3)	−0.0045 (2)	−0.0036 (2)	−0.0027 (2)
Cl4	0.0327 (3)	0.0419 (3)	0.0433 (3)	0.0050 (2)	0.0105 (2)	0.0109 (3)
N1	0.0336 (11)	0.0330 (10)	0.0270 (10)	−0.0009 (8)	0.0001 (8)	−0.0034 (8)
N2	0.0328 (11)	0.0316 (10)	0.0320 (10)	−0.0065 (8)	0.0030 (8)	−0.0065 (8)
C1	0.0362 (13)	0.0354 (12)	0.0361 (13)	0.0073 (10)	0.0022 (10)	0.0015 (10)
C2	0.0417 (14)	0.0238 (10)	0.0381 (13)	−0.0017 (9)	0.0097 (10)	−0.0025 (9)
C3	0.0304 (12)	0.0330 (11)	0.0397 (13)	0.0019 (9)	−0.0031 (10)	−0.0061 (10)
C4	0.0366 (12)	0.0252 (10)	0.0333 (12)	0.0016 (9)	−0.0009 (9)	−0.0026 (9)
O1W	0.0435 (13)	0.0692 (14)	0.0818 (18)	0.0050 (11)	0.0183 (12)	0.0334 (13)

### Geometric parameters (Å, °)

**Table:**

Cd1—Cl3	2.4418 (6)	C1—C2	1.510 (4)
Cd1—Cl4	2.4435 (6)	C1—H5	0.9700
Cd1—Cl1	2.4712 (6)	C1—H6	0.9700
Cd1—Cl2	2.4891 (7)	C2—H7	0.9700
N1—C1	1.488 (3)	C2—H8	0.9700
N1—C4	1.497 (3)	C3—C4	1.497 (3)
N1—H1	0.93 (3)	C3—H9	0.9700
N1—H2	0.89 (3)	C3—H10	0.9700
N2—C2	1.482 (3)	C4—H11	0.9700
N2—C3	1.491 (3)	C4—H12	0.9700
N2—H3	0.89 (3)	O1W—H1W	0.84
N2—H4	0.81 (3)	O1W—H2W	0.85
			
Cl3—Cd1—Cl4	115.19 (2)	C2—C1—H6	109.5
Cl3—Cd1—Cl1	108.13 (2)	H5—C1—H6	108.1
Cl4—Cd1—Cl1	115.38 (2)	N2—C2—C1	110.57 (19)
Cl3—Cd1—Cl2	110.89 (2)	N2—C2—H7	109.5
Cl4—Cd1—Cl2	103.07 (2)	C1—C2—H7	109.5
Cl1—Cd1—Cl2	103.42 (2)	N2—C2—H8	109.5
C1—N1—C4	111.06 (18)	C1—C2—H8	109.5
C1—N1—H2	106 (2)	H7—C2—H8	108.1
C4—N1—H2	111 (2)	N2—C3—C4	110.14 (19)
C1—N1—H1	107.8 (18)	N2—C3—H9	109.6
C4—N1—H1	111.2 (19)	C4—C3—H9	109.6
H1—N1—H2	110 (3)	N2—C3—H10	109.6
C2—N2—C3	111.28 (19)	C4—C3—H10	109.6
C2—N2—H3	113 (2)	H9—C3—H10	108.1
C3—N2—H3	104.5 (18)	C3—C4—N1	110.45 (19)
C2—N2—H4	110 (2)	C3—C4—H11	109.6
C3—N2—H4	106 (2)	N1—C4—H11	109.6
H3—N2—H4	112 (3)	C3—C4—H12	109.6
N1—C1—C2	110.8 (2)	N1—C4—H12	109.6
N1—C1—H5	109.5	H11—C4—H12	108.1
C2—C1—H5	109.5	H1W—O1W—H2W	116
N1—C1—H6	109.5		

### Hydrogen-bond geometry (Å, °)

**Table:**

*D*—H···*A*	*D*—H	H···*A*	*D*···*A*	*D*—H···*A*
N1—H1···Cl1^i^	0.93 (3)	2.35 (3)	3.254 (2)	164 (3)
N1—H2···Cl3	0.89 (3)	2.41 (4)	3.155 (2)	141 (3)
N2—H3···O1W	0.89 (3)	1.93 (3)	2.808 (3)	167 (3)
N2—H4···Cl4^ii^	0.81 (3)	2.46 (3)	3.190 (2)	151 (3)
O1W—H1W···Cl2^iii^	0.84	2.44	3.267 (3)	168
O1W—H2W···Cl4^iv^	0.85	2.54	3.304 (2)	150

Symmetry codes: (i) −*x*+1, *y*+1/2, −*z*+1/2; (ii) *x*+1, −*y*+1/2, *z*+1/2; (iii) −*x*, −*y*, −*z*+1; (iv) *x*, −*y*+1/2, *z*+1/2.
